# Impact Mechanism of New Urbanization on Environmental Pollution: Empirical Analysis Based on Spatial Panel Model

**DOI:** 10.3389/fpubh.2022.928100

**Published:** 2022-07-11

**Authors:** Yi Zhang, Qihua Cai

**Affiliations:** School of Business, Zhengzhou University, Zhengzhou, China

**Keywords:** new urbanization, environmental pollution, spatial Durbin model, effect, mechanism analysis

## Abstract

Traditional urbanization has stimulated economic growth. Meanwhile, it has damaged the natural environment. China has initiated new urbanization to resolve this dilemma. This paper aims to clarify the relationship between new urbanization and environmental pollution and prove new urbanization's superiority in containing environmental pollution. Thus, this paper adopts the static and dynamic spatial Durbin and mediating effect models to estimate the environmental pollution control mechanism of the new urbanization, using the panel data collected from 285 prefecture-level cities in China from 2003 to 2018. Findings show that: (1) Environmental pollution has time inertia and spatial spillover effect. The degree of pollution in an area is related to the environmental quality in the earlier stage and the surrounding areas. (2) The role of new urbanization in containing environmental pollution can take effect in the long run. In the short term, population urbanization can restrain the environmental pollution of both local and surrounding cities. (3) Heterogeneity analysis shows that the higher the level of environmental pollution, the greater the impact of new urbanization on environmental pollution. (4) Mediating effect test shows that technological effect and industrial structure upgrading are two important channels for new urbanization to reduce environmental pollution. (5) Threshold effect test shows that the inhibition effect of new urbanization on environmental pollution is gradually enhanced after crossing the threshold.

## Introduction

Urbanization and environmental pollution have always been essential issues for developing countries (1, 2). Although urbanization can accelerate economic development (3), it also leads to the loss of cultivated land (4) and an increase in pollution emissions (5). Since the reform and opening up, the population has moved freely between cities and villages, rapidly raising urbanization in China (6). It is the traditional urbanization that focuses on population migration. With the continuous improvement of the urbanization level, China's economic development level is also rising. However, the rapid traditional urbanization has led to “urban diseases” (7). Environmental pollution such as water pollution, air pollution, and land pollution is becoming more and more serious (8–11). Environmental pollution reduces the quality of life (1) and restricts economic growth (12, 13). In order to solve these problems, China has transformed into new urbanization.

In 2003, the report of the 16th National Congress of the Communist Party of China put forward the idea of “taking the road of urbanization with Chinese characteristics”, which means the emergence of new urbanization. In 2007, the report of the 17th National Congress of the Communist Party of China put forward the guiding ideology and construction path of new urbanization. In 2012, the report of the 18th Party Congress raised the new urbanization to a strategic level. In 2014, the “National New Urbanization Plan (2014-2020)” was officially released, which marked that new urbanization entered the stage of comprehensive construction. In 2021, China's urbanization rate reached 64.7%, which indicates that new urbanization has entered the middle and late stages of rapid development. New urbanization involves the transformation of population, economy, society, and green. Its interaction with environmental pollution will be complicated. Therefore, the primary purpose of this paper is to clarify the relationship between new urbanization and environmental pollution and then analyze how new urbanization affects environmental pollution.

Environmental pollution affects the sustainability of economic development and human health (14–16). Innovation, environmental supervision, and urbanization are essential factors affecting environmental pollution. Advanced industrial technology can help enterprises make environmental-friendly decisions (17), thus improving innovation efficiency and inhibiting the ecological footprint (18). Technological innovations can incentivize cities to improve energy efficiency and replace fossil fuels with clean energy, reducing pollution emissions (19–21). Taxes can also encourage enterprises to use clean energy and reduce carbon emissions (22). The cost of environmental supervision can increase the R&D expenditure of enterprises (23), thus improving productivity.

Scholars have two main views on the relationship between urbanization and environmental pollution. The first view is that the relationship between urbanization and environmental pollution is linear. Urbanization can increase environmental pollution by increasing population density, reducing air diffusion capacity, and increasing domestic sewage discharge (8, 24–26). Urbanization can also reduce environmental pollution by saving public space, increasing residents' income, and promoting the construction of smart cities (27–29). The second view is that the relationship between urbanization and environmental pollution is nonlinear. The impact of urbanization on environmental pollution will change with the degree of regional and economic development (30). The Environmental Kuznets curve may have different forms in different regions (31). However, this U-shaped trend is uncertain (32, 33). In addition, environmental pollution can produce cross-border pollution through spatial diffusion (34). Therefore, many scholars have discussed the spatial spillover effects of key variables when discussing environmental pollution (35, 36). Thus, this paper uses a spatial econometric model to discuss the spatial correlation effect of environmental pollution.

There are some shortcomings in the existing research. First, most scholars have discussed the relationship between traditional urbanization and environmental pollution. However, few have discussed the relationship between new urbanization and environmental pollution. Second, when most papers discuss the spatial correlation of environmental pollution, they lack attention to the time lag effect. Third, there may be a complex interaction between new urbanization and environmental pollution, and its specific mechanism must be explored.

The research contribution of this paper has three aspects. Firstly, the inhibition effect and spatial spillover effect of new urbanization on environmental pollution are investigated based on establishing new urbanization indicators. Secondly, the superposition effect of environmental pollution in time and space is analyzed based on the spatial econometric model. Through effect decomposition, the effects of new urbanization and its sub-dimensions on environmental pollution are discussed from long-term and short-term perspectives. Finally, using the mediating effect and threshold effect model, the specific impact mechanism of new urbanization on environmental pollution is studied.

## Theoretical Analysis and Hypothesis

New urbanization can directly affect environmental pollution, and it can also indirectly affect environmental pollution by affecting regional economic characteristics.

### Direct Effect

Promoting new urbanization involves the multi-dimensional transformation of population, economy, society, and green. The impacts of each dimension on environmental pollution are as follows:

Population urbanization is mainly reflected in the migration and concentration of rural populations to cities and towns. Cities with high population density have economies of scale in the supply of public infrastructure, which can reduce energy consumption and pollution emissions (36). The spatial concentration of the population has further promoted the exchange of knowledge, information, and technology and strengthened social relations. Thereby improving the efficiency of enterprise ecological innovation (37).

Economic urbanization gives new urbanization more attention to intensive economic development. This mode can improve environmental quality and reduce pollution emissions (38).

Social urbanization emphasizes the remodeling of lifestyle and the change of quality of life (39). It has enhanced the public's willingness to participate in environmental protection and strengthened the concept of sustainable economic development. Environmental pollution has negative externalities. With the increase of public participation in geographically adjacent areas, the local environmental quality can be improved (40).

The concept of green development guides green urbanization to realize the coordinated development of the economy, society, and ecological environment. By improving the environmental supervision system, the government has increased the cost of enterprise pollution discharge (22); with extensive social supervision, the public has adopted a green lifestyle (41). All these measures have reduced environmental pollution. Urbanization also actively influences environmental pollution control by promoting green technologies (42). In summary, this paper puts forward hypothesis 1.

Hypothesis 1: New urbanization can reduce environmental pollution.

### Indirect Effect

New urbanization can bring technological innovation and industrial structure upgrading, thus reducing the environmental pollution.

New urbanization has promoted the agglomeration of population, capital, technology, and other factors, as well as the agglomeration of enterprises with high productivity, high added value, and high technology level. It speeds up the dissemination of knowledge and the sharing of resources, promotes the communication between industries and the learning between enterprises, and makes technological innovation easier to realize (43), thus producing the technological effect. This effect can restrain environmental pollution from two aspects. First, technological innovation can reduce the use of high pollution, high energy consumption, and high emission energy by enterprises (44, 45). Second, the spillover of technological innovation can reduce the cost of environmental pollution prevention for local enterprises and surrounding enterprises, thus reducing pollution emissions.

New urbanization has optimized the industrial structure, promoted industrial integration, and upgraded the industrial structure. The industrial structure upgrading has adjusted the proportional relationship between industries, changed the demand structure of industries for energy, and made high energy-consuming enterprises withdraw from the market. It also has improved the adoption level of green technology and realized the green transformation of enterprises, thus reducing pollution emissions (46). In summary, this paper puts forward hypothesis 2.

Hypothesis 2: New urbanization can restrain environmental pollution through technological effects and industrial structure upgrading.

## Research Design

### Variables

#### New Urbanization

New urbanization is an organic whole involving urban development factors such as population, economy, society, and green. Scholars have yet to agree on constructing a new urbanization index system. According to the connotation of new urbanization, this paper constructs the index system of new urbanization from four dimensions: population, economy, society, and green, combined with the existing research results (10, 47–49). This paper uses the entropy method to measure new urbanization according to this index system.

Population urbanization mainly includes two aspects: population mobility and employment level. Therefore, this paper uses population density and urbanization rate to describe population mobility and uses the proportion of employees in the secondary industry, the proportion of employees in the tertiary industry, and the proportion of urban employment density to reflect the employment level.

Economic urbanization mainly includes residents' income and consumption, industrial structure, and investment level. Therefore, this paper uses the average wage of employees, the per capita savings balance of financial institutions, and the retail sales of social consumer goods to describe residents' income and consumption, and uses the proportion of secondary industry to GDP, the proportion of tertiary industry to GDP and the investment of fixed assets per unit GDP to reflect the industrial structure and investment situation.

Social urbanization mainly includes three aspects: equalizing public services, improving quality of life, and integrating urban and rural areas. Therefore, this paper uses the number of hospital beds per capita, the number of buses per capita, and the highway density to reflect the level of equalization of public services, the urban per capita disposable income, the per capita paved road area, the per capita library collection, and the number of Internet broadband users to describe the quality of life of residents, and the proportion of urban and rural per capita disposable income to reflect the development degree of urban–rural integration.

Green urbanization mainly includes green space construction and waste disposal. Therefore, this paper uses the green coverage rate and per capita green area of the built-up area to describe the level of green space construction and uses the domestic garbage treatment rate and the comprehensive utilization rate of industrial solid waste to reflect the waste treatment situation.

The index system of new urbanization is shown in Table 1.

**Table 1:** The index system of new urbanization.

**System-level**	**First-level indicators**	**Second-level indicators**	**Unit**
New urbanization	Population urbanization	Urban employment density	%
		Population density	person/km^2^
		Proportion of employees in the secondary industry	%
		Proportion of employees in the tertiary industry	%
		Urbanization rate	%
	Economic urbanization	Per capita retail sales of social consumer goods	yuan
		Ratio of secondary industry to GDP	%
		Ratio of tertiary industry to GDP	%
		Average wage of staff and workers	yuan
		Per capita savings deposit balance of financial institutions	yuan
		Investment of fixed assets per unit of GDP	yuan
	Social urbanization	Number of beds in medical facilities per 10,000 population	bed/10,000
		Per capita amount of library collections	volume/person
		Urban per capita disposable income	yuan
		Highway density	*km*/*km*^2^
		Number of public transport vehicles per 10,000 population	vehicle/10,000
		Ratio of the disposable income of urban and rural residents	%
		Number of internet broadband access users	household
		Per capita area of paved roads	*m*^2^/person
	Green urbanization	Green coverage rate of completed area	%
		Per capita green area	park area/city population
		Treatment rate of consumption wastes	%
		Comprehensive utilization rate of industrial solid wastes	%

#### Environmental Pollution

According to the existing literature (10, 50) and the diversity of pollutants, this paper uses the emissions of sulfur dioxide, industrial wastewater, and industrial smoke (powder) dust to construct an environmental pollution index system. This paper uses the entropy method to measure environmental pollution based on this index system.

#### Control Variables

(1) The level of economic development (LnGDP). It is expressed in the logarithmic form of GDP per capita of each city. According to the environmental Kuznets hypothesis, the relationship between economic growth and environmental pollution may be inverted U-shaped. In order to reflect the nonlinear relationship between economic level and environmental pollution, this paper introduces the square term of economic development level (lnGDP2).
(2) Whether the high-speed rail is opened (HR). At present, roads are an indispensable means of transportation in China. The construction of a high-speed railway is different from the construction of an expressway network. The distribution of the high-speed rail network is limited by the characteristics of the city and the government planning. It is difficult to achieve regional balance and equalization of public services. However, the construction of a high-speed railway network increases the density of the transportation network, improves the efficiency and frequency of resource transportation, and reduces urban congestion. It helps to reduce the emissions per unit of transportation (51, 52). Therefore, this paper introduces a dummy variable, whether the high-speed rail is open (HR), to describe the construction of urban high-speed rail.
(3) Natural growth (NGR). The population growth rate increases the potential for economic growth. However, it also increases energy demand and consumption.
(4) Open to the outside world (OPEN). It can affect the emission of pollutants in the production process of domestic production enterprises by introducing foreign advanced cleaning technology. This paper uses GDP's total import and export percentage to measure opening up (53).

#### Mediating Variables

Technology effect and industrial structure upgrading are two channels for new urbanization to affect environmental pollution. Considering that the number of patents is the most commonly used index to measure technological innovation, this paper uses the number of three patents accepted in cities to measure the technological effect (TECH).

This paper uses the method of the existing literature (54) to measure industrial structure upgrading (INS). A group of three-dimensional space vector *X*_0_ = (*X*_1,0_, *X*_2,0_, *X*_3,0_) is constructed by taking the added value of the three industries as a proportion of GDP. Then, based on the vector groups *X*_1_ = (1, 0, 0), *X*_2_ = (0, 1, 0), *X*_3_ = (0, 0, 1), calculate the included angle θ_*j*_, (*j* = 1, 2, 3) between the three vectors and *X*_0_, respectively.


(1) $$\begin{array}{l} \theta_{j}=\arccos\left(\frac{\overset{3}{\underset{i=1}{\sum }}\left(x_{i,j}\;\cdot \;x_{i,0}\right)}{\left(\overset{3}{\underset{i=1}{\sum }}{\left(x_{i,j}^{2}\right)}^{1/2}\;\cdot \;\overset{3}{\underset{i=1}{\sum }}{\left(x_{i,0}^{2}\right)}^{1/2}\right)}\right) \end{array}$$

The industrial structure upgrading (INS) estimation method is as follows:


(2) $$\begin{array}{l} W=\overset{3}{\underset{k=1}{\sum }}\overset{k}{\underset{j=1}{\sum }}\theta_{j} \end{array}$$

The greater the value of W, the higher the degree of industrial structure upgrading.

### Data

The cities involved in this paper are all prefecture-level cities. Chaohu City, Bijie City, and other cities that have undergone zoning adjustments and some cities with incomplete data are excluded. Finally, the balanced panel data of 285 prefecture-level cities in China from 2003 to 2018 are selected as the research sample. The specific data come from the China Urban Statistical Yearbook, China Environmental Statistical Yearbook, China Urban and Industrial Innovation Report, and statistical yearbooks and statistical bulletins of all provinces in China. Table 2 shows descriptive statistics.

**Table 2:** Descriptive statistics.

**Variable**	**Obs**	**Mean**	**Std. Dev**	**Min**	**Max**
EP	4560	0.037	0.041	0.001	0.454
NUR	4560	0.113	0.029	0.053	0.558
HR	4560	0.318	0.466	0	1
LnPGDP	4560	10.063	0.942	6.638	13.185
LnPGDP2	4560	102.160	19.090	44.060	173.846
OPEN	4560	0.251	0.881	0.000	29.327
NGR	4560	5.766	5.455	−16.64	113
TECH	4560	0.443	1.326	0.003	21.442
INS	4560	6.446	0.385	5.432	7.993

We chose the beginning of 2003 and the end of 2018 as the research period. On the one hand, the 16th National Congress of the Communist Party of China (CPC) proposed “take the road of urbanization with Chinese characteristics”, meaning the embryonic form of new urbanization emerged. Since then, the whole country has started to promote new urbanization independently. On the other hand, there are three reasons for choosing 2018 as the end time: First, the statistical methods of the China Urban Statistical Yearbook in 2019 have changed, resulting in some basic data of the indicators constructed in this paper being unavailable. Second, many cities are involved in this study, taking prefecture-level cities as the basic unit. However, the construction environmental pollution index of many provinces in 2019 has not been updated (Anhui, Shanxi, etc.), which will affect the index calculation of essential variables in this paper. Third, new urbanization is a long-term strategy. The national new urbanization plan (2014-2020) indicates that the strategic priorities of new urbanization will not be significantly adjusted in recent years, the key points of new urbanization construction in 2019 still emphasize the collaborative, green, and intensive development of the economy and society, and the constructed indicator system has not changed significantly. In addition, due to the outbreak of COVID-19 at the end of 2019, the shutdown of factories affected normal economic life. It resulted in an abnormal pollution situation in 2020 compared with previous ones, so we used 2018 as the end of the sample period.

The final scores of critical variables are calculated by the entropy method. The entropy method calculation process is as follows:

Normalize the indicators, and the positive indicators are:


(3) $$\begin{array}{l} {\overset{-}{x}}_{ij}=\frac{\alpha_{ij-\min\left(\alpha_{ij}\right)}}{\max\;(\alpha_{ij})-\min\;(\alpha_{ij})} \end{array}$$

The negative indicators are:


(4) $$\begin{array}{l} {\overset{-}{x}}_{ij}=\frac{\max\;(\alpha_{ij})-\alpha_{ij}}{\max\;(\alpha_{ij})-\min\;(\alpha_{ij})} \end{array}$$

In the *j* index, the proportion of the data of the *i*_th_ city is:


(5) $$\begin{array}{l} p_{ij}=\frac{x_{ij}}{\overset{m}{\underset{i=1}{\sum }}x_{ij}} \end{array}$$

Entropy value of item *j* index:


(6) $$\begin{array}{l} e_{j}=-\frac{\overset{m}{\underset{i=1}{\sum }}\left(p_{ij}\ln\;p_{ij}\right)}{\ln\;m} \end{array}$$

The weight of item *j* index:


(7) $$\begin{array}{l} w_{j}=\frac{1-ej}{\overset{n}{\underset{j=1}{\sum }}\left(1-e_{j}\right)} \end{array}$$

### Methods

#### Spatial Correlation

We use Moran's I to analyze the spatial correlation between environmental pollution and new urbanization. The formula is:


(8) $$\begin{array}{l} I=\frac{\text{n}\overset{n}{\underset{i=1}{\sum }}\overset{n}{\underset{j=1}{\sum }}w_{ij}\;(x_{\text{i}}-\overset{-}{x})\;\left(x_{j}-\overset{-}{x}\right)}{\overset{n}{\underset{\text{i}=1}{\sum }}\overset{n}{\underset{j=1}{\sum }}w_{ij}\overset{n}{\underset{i=1}{\sum }}{\left(x_{\text{i}}-\overset{-}{x}\right)}^{2}} \end{array}$$

where *n* denotes the number of research samples and *W*_*ij*_ is the spatial weight matrix. *I* denotes the global Moran index, and its index value ranges from −1 to 1. If *I* is greater than 0, it means a positive spatial correlation between variables. Otherwise, there is a negative spatial correlation between variables. The closer *I* is to 1, the greater the absolute value of global Moran's I and the stronger the spatial correlation (40, 55). Geographic distance is an essential factor affecting the frequency of economic ties. Therefore, referring to the common practices of previous scholars (14, 40), we use a geographical distance weight matrix (W_1_) to estimate the spatial effect, and the robustness test is performed using an economic matrix (W_2_) and an adjacency matrix (W_3_).


(9) $$\begin{array}{l} W_{1}=\{\begin{array}{c} \frac{1}{d_{ij}},\;if\;i\neq j\\ 0\;,\;if\;i=j \end{array} \end{array}$$

(10) $$\begin{array}{l} W_{2}=\{\begin{array}{c} \frac{1}{\left|\bar{GDP_{i}}-\bar{GDP_{j}}\right|},\;if\;i\neq j\\ 0\;,\;if\;i=j \end{array} \end{array}$$

(11) $$\begin{array}{l} W_{3}=\{\begin{array}{c} 1\;,\;if\;i\neq j\\ 0\;,\;if\;i=j \end{array} \end{array}$$

*d*_*ij*_ is the geographical distance between city *I* and city *J*, calculated according to the longitude and latitude of the city. In this paper, the reciprocal of the distance between two cities is used as the spatial weight matrix, which can reflect the proximity of two regions and reflect the convenience of transportation. The spatial correlation test results of environmental pollution and the new urbanization index in China's sample areas are shown in Table 4.

#### Model Selection

Before the spatial econometric analysis, the model's applicability needs to be tested. Results as shown in Table 3, the LM and LR tests' statistics are significant at 1% level, which indicates that it is necessary to use the spatial measurement method for analysis. Compared with spatial lag and error models, the spatial Durbin model has more advantages in alleviating estimation errors (35). The Hausman test shows that this paper must control the time fixed effect and individual fixed effect in the spatial Durbin model. The statistics of the Wald test are significant at the level of 1%, which shows that the spatial Durbin model cannot be degraded into spatial lag and spatial error model. To sum up, this paper chooses the spatial Durbin model for analysis.

**Table 3:** Panel-section dependence test.

**Test**	**Statistical value**	***P*-value**
Robust LM-lag	228.762	0.000
Robust LM-error	1686.338	0.000
Hausman	55.70	0.000
LR-SDM-SEM	20.68	0.002
LR-SDM-SAR	16.88	0.010
Wald-Spatial-lag	20.72	0.002
Wald-Spatial-error	16.88	0.010

The specific form of the model is set as follows:


(12) $$\begin{array}{l} EP_{it}=\alpha_{0}+\beta_{0}\overset{n}{\underset{j=1}{\sum }}w_{ij}EP_{it}+\beta_{1}NUR_{it}+\beta_{2}x_{it}\\ +\beta_{3}\overset{n}{\underset{j=1}{\sum }}w_{ij}NUR_{jt}+\beta_{4}\overset{n}{\underset{j=1}{\sum }}w_{ij}x_{jt}+\mu_{i}+\lambda_{i}+\varepsilon_{it} \end{array}$$

In the above formula, *EP*_*it*_, *NUR*_*it*_, *X*_*it*_ represent explained variables, explanatory variables, and a series of control variables; *W*_*ij*_ indicates the spatial weight matrix; β_0_, β_3_, β_4_ represent the spatial lag effect coefficient of explained, explanatory, and control variables. μ_*i*_ and λ_*i*_ indicate the individual and time effects, respectively. ε_*it*_ signifies the idiosyncratic error term.

Considering that the change in pollution discharge is a dynamic process, it will be adjusted with the change in urbanization strategy. In order to explore the dynamic effect of environmental pollution, based on the above measurement model, the lagging term of environmental pollution is added to form a dynamic panel model. The specific model forms are as follows:


(13) $$\begin{array}{l} EP_{it}=\alpha_{1}+\tau EP_{i,t-1}+\gamma_{0}\overset{n}{\underset{j=1}{\sum }}w_{ij}EP_{it}+\gamma_{1}NUR_{it}+\gamma_{2}x_{it}\\ +\gamma_{3}\overset{n}{\underset{j=1}{\sum }}w_{ij}EP_{ij\left(t-1\right)}+\gamma_{4}\overset{n}{\underset{j=1}{\sum }}w_{ij}NUR_{jt}\\ +\gamma_{5}\overset{n}{\underset{j=1}{\sum }}w_{ij}x_{jt}+\mu_{i}+\lambda_{i}+\varepsilon_{it} \end{array}$$

In the formula, τ* and γ*_3_ indicate the time delay effect coefficient and space-time double lag effect coefficient of explanatory variables. Other variables and coefficients are similar to those in Equations (12).

#### Mediating Effect Model

In order to solve the mechanism of new urbanization to restrain environmental pollution, this paper draws lessons from Baron Kenny's three-step causality method (56). Based on the spatial Durbin model, construct the mediating effect model for regression:


(14) $$\begin{array}{l} EP_{it}=\alpha_{2}+\rho_{0}\overset{n}{\underset{j=1}{\sum }}w_{ij}EP_{it}+\rho_{1}NUR_{it}+\rho_{2}x_{it}\\ +\rho_{3}\overset{n}{\underset{j=1}{\sum }}w_{ij}NUR_{jt}+\rho_{4}\overset{n}{\underset{j=1}{\sum }}w_{ij}x_{jt}\\ +\mu_{i}+\lambda_{i}+\varepsilon_{it} \end{array}$$

(15) $$\begin{array}{l} M_{ij}=\alpha_{2}^{\prime }+\rho_{0}^{\prime }\overset{n}{\underset{j=1}{\sum }}w_{ij}M_{ij}+\rho_{1}^{\prime }NUR_{it}+\rho_{2}^{\prime }x_{it}\\ +\rho_{3}^{\prime }\overset{n}{\underset{j=1}{\sum }}w_{ij}NUR_{jt}+\rho_{4}^{\prime }\overset{n}{\underset{j=1}{\sum }}w_{ij}x_{jt}\\ +\mu_{i}+\lambda_{i}+\varepsilon_{it} \end{array}$$

(16) $$\begin{array}{l} EP_{it}=\alpha_{2}^{\prime \prime }+\rho_{0}^{\prime \prime }\overset{n}{\underset{j=1}{\sum }}w_{ij}EP_{it}+\rho_{1}^{\prime \prime }NUR_{it}\\ +\rho_{2}^{\prime \prime }x_{it}+\rho_{3}^{\prime \prime }\overset{n}{\underset{j=1}{\sum }}w_{ij}NUR_{jt}+\rho_{4}^{\prime \prime }\overset{n}{\underset{j=1}{\sum }}w_{ij}x_{jt}\\ +\rho_{5}M_{ij}+\rho_{6}\overset{n}{\underset{j=1}{\sum }}w_{ij}M_{ij}+\mu_{i}+\lambda_{i}+\varepsilon_{it} \end{array}$$

In Equations (15) and (16), the *M*_*ij*_ is an intermediary variable, including technological effect and industrial structure upgrading. Other variables and coefficients are similar to the previous formula.

## Results

### Spatial Autocorrelation Analysis

#### Global Autocorrelation

Considering that there are significant regional differences between environmental pollution and new urbanization development in cities in China, this paper uses the Moran index tests, the spatial correlation of critical variables.

According to Table 4, the Moran index values for each year are significantly greater than 0. It indicates apparent spatial positive correlations and spatial clustering phenomena between environmental pollution and new urbanization. Therefore, it is logical to consider the spatial correlation between environmental pollution and new urbanization in the empirical process.

**Table 4:** Global correlation test—Moran index.

**Year**	**EP**			**NUR**		
	***I***	***Z***	***P***	***I***	***Z***	***P***
2003	0.018	4.376	0.000	0.092	18.894	0.000
2004	0.026	5.987	0.000	0.073	15.132	0.000
2005	0.028	6.324	0.000	0.080	16.681	0.000
2006	0.032	7.241	0.000	0.077	16.244	0.000
2007	0.027	6.223	0.000	0.080	16.904	0.000
2008	0.029	6.531	0.000	0.083	17.586	0.000
2009	0.031	6.952	0.000	0.079	16.744	0.000
2010	0.041	8.868	0.000	0.049	12.607	0.000
2011	0.028	6.330	0.000	0.064	13.464	0.000
2012	0.030	6.851	0.000	0.082	17.003	0.000
2013	0.045	9.645	0.000	0.091	18.711	0.000
2014	0.053	11.362	0.000	0.083	17.166	0.000
2015	0.053	11.257	0.000	0.085	17.705	0.000
2016	0.064	13.518	0.000	0.089	18.456	0.000
2017	0.075	15.698	0.000	0.093	19.239	0.000
2018	0.044	10.293	0.000	0.089	18.345	0.000

#### Local Autocorrelation

**Figure 1:**
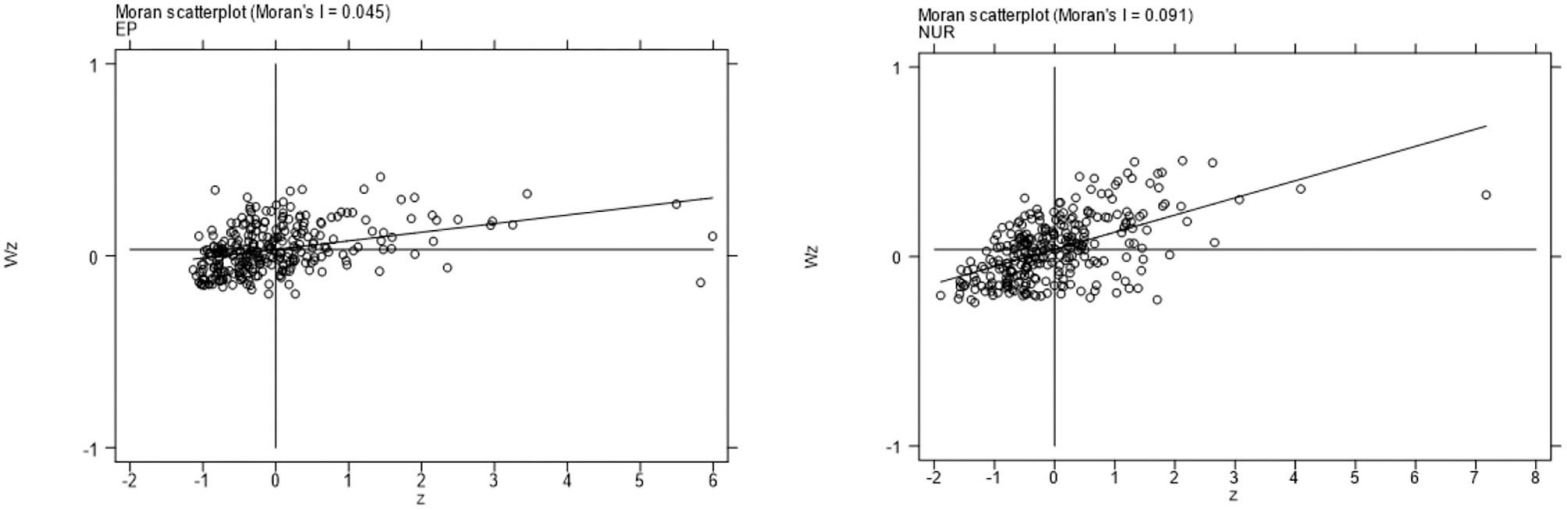
Scatter plot of the Moran index of China's environmental pollution and new urbanization in 2013.

Although the Moran index can reflect the spatial agglomeration characteristics of the overall distribution of each region, it can only measure the average degree of spatial differences among regions. It cannot distinguish between high-value agglomeration and low-value agglomeration of spatial patterns. The Moran scatter diagram can make up for this defect. Figure 1 takes the Moran scatter diagram of environmental pollution and new urbanization in 2013 as an example. The results show that most of the scatter plots of the Moran index are concentrated in the first quadrant (H-H region) and the third quadrant (L-L region). It is reasonable to discuss the inhibitory effect of new urbanization on environmental pollution under the spatial background.

### Empirical Analysis

#### Baseline Regression Model

Table 5 reports the estimated results of new urbanization levels and environmental pollution in 285 prefecture-level cities in China from 2003 to 2018. For the convenience of comparative analysis, models (1) and (2) are the estimation results of the random effect of new urbanization on environmental pollution and the double fixed effect of time and individual without considering the spatial effect. When the spatial correlation is not considered, the regression coefficient of new urbanization to environmental pollution is significantly negative. It indicates that new urbanization has an inhibitory effect on environmental pollution.

**Table 5:** Baseline regression model.

**Variables**	**(1)**	**(2)**	**(3)**		**(4)**	
			**Main**	**Wx**	**Main**	**Wx**
L.EP					0.317^***^	31.162^***^
					(0.014)	(0.183)
NUR	−0.245^***^	−0.189^**^	−0.172^***^	−0.231	−0.087^**^	16.230^***^
	(0.065)	(0.084)	(0.043)	(0.402)	(0.042)	(0.415)
HR	0.089	−0.005^***^	−0.006^***^	0.015	0.004^***^	−0.226^***^
	(0.133)	(0.002)	(0.001)	(0.013)	(0.001)	(0.012)
LnPGDP	−0.012^***^	0.024^*^	0.041^***^	−0.212^***^	0.056^***^	0.855^***^
	(0.002)	(0.013)	(0.008)	(0.060)	(0.008)	(0.063)
LnPGDP2	0.031^***^	−0.001^*^	−0.002^***^	0.011^***^	−0.003^***^	−0.027^***^
	(0.011)	(0.001)	(0.000)	(0.003)	(0.000)	(0.003)
OPEN	−0.001^**^	0.000	0.000	0.001	−0.001	−0.051^***^
	(0.001)	(0.000)	(0.000)	(0.006)	(0.000)	(0.006)
NGR	0.000	0.000^*^	0.000	−0.000	−0.000^***^	−0.003^***^
	(0.000)	(0.000)	(0.000)	(0.001)	(0.000)	(0.001)
Constant	0.000^**^	−0.064				
	(0.000)	(0.064)				
ρ			0.575^***^		9.032^***^	
			(0.084)		(0.101)	
Year effect		YES	YES	YES	YES	YES
Individual effect		YES	YES	YES	YES	YES
City	285	285	285	285	285	285

*Standard errors in parentheses*.

*** *p < 0.01*,

** *p < 0.05*,

* *p < 0.1*.

Affected by the geographical environment (natural activities such as the atmosphere and rivers) and regional economic activities (57), pollution emissions from a local area can spread to the surrounding area. However, traditional model regression cannot reflect the spatial correlation of key variables among regions. Therefore, this paper continues to use the spatial econometric model for testing.

Based on the geographical distance weight matrix, we establish a spatial Durbin model with time and individual fixed effects to analyze the impact of new urbanization on environmental pollution. The samples studied in this paper cover a wide geographical range. The individual fixed effect eliminates the influence of short-term factors that do not change with time, such as cities' geographical location and policy. Time-fixed effects control macroeconomic factors and policy changes affecting all cities in a specific year.

In Table 5, the coefficient of the spatial lag term is negative. It has passed the significance test at 1% level, meaning that environmental pollution has a positive spatial spillover effect. The spatial spillover effect may be that the environmental protection policies of cities with similar geographical locations may be similar, and the pollutants will spread to other regions under the influence of geographical factors. The impact of new urbanization on pollution emissions is negative significantly at 1% level. It means that new urbanization can effectively inhibit environmental pollution in cities. Hypothesis 1 has been verified.

The static spatial panel model only contains the spatial lag effect of the sample area. However, environmental pollution is a dynamic process that accumulates continuously. Hence, it is necessary to consider the time lag term of environmental pollution. Model (4) shows the regression result of the spatial dynamic Durbin model with double fixed effects. The spatial lag term is significant at the significance level of 1%. The coefficient is larger than that estimated by the static model, which indicates that the static model underestimated the spatial effect of environmental pollution. It confirmed that the environmental pollution changes are time-dependent and path-dependent. In the early stage, the environmental pollution problem worsened the city's ecological environment, increased the difficulty of environmental pollution control in this period, and formed a negative demonstration for the surrounding areas.

In order to reflect the impact of the main variables on the surrounding environmental pollution in more detail, we estimated the direct, indirect, and total effects of new urbanization on environmental pollution. The results are shown in Table 6.

**Table 6:** Direct and indirect effects of baseline regression.

**Variables**	**Direct**	**Indirect**	**Total**
	**effect**	**effect**	**effect**
NUR	−0.172^***^	−0.780	−0.953
	(0.044)	(1.025)	(1.025)
HR	−0.006^***^	0.026	0.020
	(0.001)	(0.029)	(0.028)
LnPGDP	0.040^***^	−0.451^***^	−0.411^***^
	(0.007)	(0.158)	(0.156)
LnPGDP2	−0.002^***^	0.023^***^	0.021^***^
	(0.000)	(0.008)	(0.008)
OPEN	0.000	0.002	0.002
	(0.000)	(0.015)	(0.015)
NGR	0.000^*^	0.000	0.000
	(0.000)	(0.002)	(0.002)

*Standard errors in parentheses*.

*** *p < 0.01*,

* *p < 0.1*.

The estimated coefficient of new urbanization on environmental pollution is −0.172. The significance test at 1% shows that new urbanization can significantly reduce local environmental pollution. However, the indirect effect did not pass the significance test, which shows that new urbanization in local areas has a limited inhibitory effect on the environmental pollution of surrounding cities.

From the control variables, the opening of high-speed rail can significantly inhibit the environmental pollution in this region. It shows that high-speed rail transportation can improve resource transportation efficiency and frequency and help alleviate urban congestion. The quadratic coefficient of the direct effect of the economy is significantly negative, which indicates an inverted U-shaped relationship between the improvement of the economic level and the environmental pollution in the local area. The reason may be that in the stage of economic take-off, the use of resources exceeds the regeneration of resources, which worsens the environmental situation. When the economy develops to a new stage, the technological and structural effects win out, and the environmental deterioration is slowed down. In addition, the increase in population growth rate will increase the economic growth potential. However, at the same time, it will also increase the energy demand and energy consumption, thus worsening the local environmental pollution situation.

#### Spatial Dynamic Durbin Model

It can be seen from the previous that new urbanization has a limited effect on the environmental pollution of surrounding cities. However, it may take some time for new urbanization to play its role, and the mitigation effect of its sub-systems on environmental pollution may change with time. In order to investigate the long-term and short-term effects of new urbanization on environmental pollution and make up for the deficiency of static spatial measurement methods and the robustness test of the previous regression results, this part uses the QML method to estimate the parameters (58). The results are shown in Table 7.

**Table 7:** Direct and indirect effects of the dynamic SDM.

**Variables**	**Short-term**			**Long-term**		
	**Direct effect**	**Indirect effect**	**Total effect**	**Direct effect**	**Indirect effect**	**Total effect**
NUR	−0.609	−1.405	−2.015^***^	−0.177^***^	−0.232^***^	−0.409^***^
	(15.953)	(15.955)	(0.055)	(0.061)	(0.060)	(0.010)
Po-Urban	−0.180^*^	−0.316^***^	−0.496^***^	−0.046^***^	−0.063^***^	−0.109^***^
	(0.092)	(0.090)	(0.016)	(0.013)	(0.012)	(0.004)
Ec-Urban	−0.199	−0.409	−0.609^***^	−0.133	0.011	−0.122^***^
	(4.412)	(4.413)	(0.024)	(0.196)	(0.196)	(0.004)
So-Urban	3.027	−5.553	−2.526^***^	−0.332^***^	−0.139^***^	−0.472^***^
	(52.712)	(52.714)	(0.035)	(0.010)	(0.008)	(0.006)
Gr-Urban	0.309	−0.214	0.095^***^	0.101^*^	−0.080	0.021^***^
	(4.705)	(4.706)	(0.007)	(0.056)	(0.056)	(0.002)
Controls	YES	YES	YES	YES	YES	YES
City	285	285	285	285	285	285

*Standard errors in parentheses*.

*** *p < 0.01*,

* *p < 0.1*.

The results show that new urbanization's long-term and short-term total effect coefficients are significant at 1%. It indicates that whether in the short term or long term, promoting new urbanization is conducive to reducing the city's pollution level and improving the environmental governance benefits. New urbanization's direct and indirect effects on environmental pollution are mainly reflected in the long term compared with the short term. It indicates that the mitigation effect of new urbanization on local and surrounding environmental pollution needs long-term transmission to be fully manifested.

The new urbanization has a limited inhibitory effect on environmental pollution in the short term. However, new urbanization can restrain environmental pollution by promoting population urbanization in the long- and short term. Population urbanization also has a spatial spillover effect. On the one hand, the concentration of the urban population facilitates the centralized management of pollutant discharge. It reduces local environmental pollution, thus reducing the diffusion of pollutants to other cities. On the other hand, inter-regional population mobility promotes knowledge and technology exchange. The inter-city learning effect can effectively promote technology spillover, making the spatial spillover effect of population urbanization significant.

In the early stage of new urbanization, cities need to carry on the past economic development mode and continue to adopt the development mode of rapid industrialization to promote urbanization. Large-scale construction, transportation, and energy consumption in urban development zones are difficult to reduce in the short term. The consumption demand for pollution-intensive products is still very significant (59). So economic urbanization does not have an obvious inhibition effect on environmental pollution in the short term. Moreover, it is not easy to build a fair and harmonious society and improve the living quality of urban residents in the short term. Suppose the government adopts more strict environmental regulations means. In that case, it may increase the cost of pollution control for enterprises and raise production costs. Therefore, social and green urbanization have limited restraining effects on environmental pollution in the short term.

In the long run, population and economic urbanization are essential factors in curbing environmental pollution. Regarding population and social urbanization, urban population density can create economic density (60), reduce the unit cost of clean energy production and public transportation services, and affect environmental quality. In the long run, the city's requirements for the living environment have increased, and environmental protection awareness has been enhanced. So the degree of social participation in environmental protection has increased, and government management's supervision has been strengthened. Economic urbanization has transformed economic development from extensive to intensive and promoted industrial structure upgrading. High-polluting enterprises have been forced out of the market, and the efficiency of economic development has been improved, which is conducive to improving environmental quality and reducing pollution emissions. In terms of green urbanization, the city's green technology upgrading and green financial system have been improved. The government has increased the green credit line of enterprises to meet the capital needs of the green industry, and enterprises have reduced the production cost of using green technology and improved the governance efficiency of pollution emissions. However, local governments in neighboring cities may compete in environmental governance, limiting the spatial spillover effects of green urbanization.

#### Heterogeneity Analysis

The above empirical results show that new urbanization development in various cities has an obvious inhibition effect on environmental pollution. Considering that the cities classified into the same area have different environmental pollution conditions, this paper uses the quantile model to analyze the inhibition effect of new urbanization in different cities on environmental pollution. The results are shown in Table 8.

**Table 8:** Quantile regression.

**Variables**	**10%**	**20%**	**30%**	**40%**	**50%**	**60%**	**70%**	**80%**	**90%**
NUR	−0.097^***^	−0.199^***^	−0.245^***^	−0.252^***^	−0.255^***^	−0.242^***^	−0.236^***^	−0.332^***^	−0.488^**^
	(0.031)	(0.049)	(0.059)	(0.062)	(0.063)	(0.066)	(0.076)	(0.113)	(0.190)
Ec-Urban	−0.047^***^	−0.091^***^	−0.108^***^	−0.111^***^	−0.110^***^	−0.101^***^	−0.108^***^	−0.145^***^	−0.255^***^
	(0.010)	(0.010)	(0.010)	(0.012)	(0.012)	(0.015)	(0.017)	(0.025)	(0.060)
Po-Urban	−0.017	−0.055^**^	−0.061^**^	−0.051^*^	−0.049^*^	−0.037	−0.042	−0.001	−0.012
	(0.015)	(0.024)	(0.027)	(0.028)	(0.030)	(0.034)	(0.044)	(0.070)	(0.111)
So-Urban	−0.052^*^	−0.109^*^	−0.145^*^	−0.155^*^	−0.169^*^	−0.190^*^	−0.218^*^	−0.348^*^	−0.504^*^
	(0.031)	(0.061)	(0.081)	(0.086)	(0.093)	(0.104)	(0.122)	(0.192)	(0.301)
Gr-Urban	−0.007^*^	−0.013^***^	−0.019^***^	−0.025^***^	−0.026^***^	−0.024^***^	−0.013^*^	−0.013	−0.027
	(0.004)	(0.004)	(0.005)	(0.005)	(0.005)	(0.006)	(0.007)	(0.009)	(0.018)
Controls	YES	YES	YES	YES	YES	YES	YES	YES	YES
City	285	285	285	285	285	285	285	285	285

*Standard errors in parentheses*.

*** *p < 0.01*,

** *p < 0.05*,

* *p < 0.1*.

It can be seen that the coefficients of the same core explanatory variable are significantly different under different quantile conditions. With the increase of quantile value, the inhibition of the new urbanization on environmental pollution gradually increases. With the aggravation of environmental pollution, the public and society's environmental protection awareness is constantly improving, so more measures and policies will be taken to control the pollution situation. Systematically, green urbanization and population urbanization have the most significant inhibition effect on the middle and low quantile areas of environmental pollution, which indicates that for cities with relatively low environmental pollution, the advantages of population agglomeration in centralized pollution control, information sharing, and technology exchange can be brought into play. The guiding and supporting role of the government and society for ecological construction can also be strengthened. However, economic and social urbanization have inhibitory effects under different levels of environmental pollution. The higher the quantile value of pollution, the more obvious the improvement effect of both on the environment. This reflects that economic development efficiency is low in heavily polluted areas. It is necessary to speed up the transformation of the economic development mode, create an intensive economic development mode, promote urban upgrading, and improve the quality of the living environment, which are effective measures to curb pollution.

#### Robustness

In order to test the stability of the above core results, this paper adopts the following methods to test the robustness: First, replace the spatial weight matrix. Second, replace the dependent variable. Finally, change the measurement of the independent variable. Results are shown in Table 9; models (1) and (2) are the regression results of economic distance and adjacency matrix, and the dependent variable of the model (3) is industrial wastewater. Model (4) uses a weighted average to measure NUR. According to the regression results in Table 9, the estimated coefficient of the direct effect of new urbanization on environmental pollution is negative. The overall results are consistent with the previous ones, which shows that the conclusions reached above are reliable. That spatial geography is not the only factor causing environmental impact. More frequent economic activities can produce closer bilateral ties.

**Table 9:** Robustness.

**Variables**	**(1)**	**(2)**	**(3)**	**(4)**
NUR	−0.197^***^	−0.192^***^	−5.684^***^	−0.084^**^
	(0.042)	(0.042)	(0.901)	(0.039)
Direct effect	−0.195^***^	−0.116^***^	−5.565^***^	−0.080^**^
	(0.043)	(0.043)	(0.922)	(0.040)
Indirect effect	0.013	0.003	32.944^*^	0.866
	(0.055)	(0.081)	(16.984)	(0.781)
Total effect	−0.183^***^	−0.114	27.379	0.786
	(0.070)	(0.090)	(16.966)	(0.779)
rho	0.059^***^	0.122^***^	0.448^***^	0.583^***^
	(0.013)	(0.020)	(0.098)	(0.083)
Controls	YES	YES	YES	YES
Year effect	YES	YES	YES	YES
Individual effect	YES	YES	YES	YES
City	285	285	285	285

*Standard errors in parentheses*.

*** *p < 0.01*,

** *p < 0.05*,

* *p < 0.1*.

## Mechanism Analysis

### Mediating Effect

According to the previous empirical results, it can be seen that new urbanization can significantly inhibit environmental pollution without adding intermediary variables.

In order to verify whether new urbanization has an intermediary effect on environmental pollution, this part further tests the mechanism using the mediating effect model.

Models (1) and (3) in Table 10 show that new urbanization has a significant role in promoting technological effects and upgrading industrial structures. Adding the intermediary variables to the previous regression, the significance of the NUR has not changed in models (2) and (4). It shows that, on the one hand, new urbanization can reduce dependence on highly polluting and energy-consuming energy. It reduces the pollution emission intensity of old energy by acting on technological effects, using high-tech to improve the resource utilization efficiency of enterprises. On the other hand, by upgrading the industrial structure, we can adjust the relative proportion of various industrial sectors in the national economy, improve the energy efficiency of enterprises, and restrict environmental pollution. Hypothesis 2 has been verified.

**Table 10:** Mediating effect.

**Variables**	**(1) TECH**	**(2) M = TECH**	**(3) INS**	**(4) M = INS**
NUR	5.515^***^	−0.005^***^	1.635^***^	−0.178^***^
	(1.486)	(0.000)	(0.392)	(0.043)
TECH		−0.140^***^		
		(0.042)		
INS				−0.003^*^
				(0.002)
HR	−0.041	−0.006^***^	0.011	−0.006^***^
	(0.039)	(0.001)	(0.010)	(0.001)
LnPGDP	−6.209^***^	0.010	0.153^**^	0.047^***^
	(0.275)	(0.008)	(0.073)	(0.008)
LnGDP2	0.324^***^	−0.000	−0.006^*^	−0.002^***^
	(0.014)	(0.000)	(0.004)	(0.000)
OPEN	−0.022^*^	−0.000	−0.004	−0.000
	(0.013)	(0.000)	(0.003)	(0.000)
NBR	0.012^***^	0.000^**^	0.001^*^	0.000^*^
	(0.003)	(0.000)	(0.001)	(0.000)
rho	0.875^***^	0.482^***^	0.365^***^	0.572^***^
	(0.030)	(0.095)	(0.109)	(0.085)
Direct effect	7.141^***^	−0.005^***^	1.702^***^	−0.003^*^
	(1.622)	(0.000)	(0.400)	(0.002)
Indirect effect	437.553^***^	−0.023^**^	17.863^***^	−0.039
	(166.257)	(0.009)	(6.728)	(0.052)
Total effect	444.694^***^	−0.028^***^	19.566^***^	−0.041
	(166.815)	(0.009)	(6.714)	(0.052)
Year effect	YES	YES	YES	YES
Individual effect	YES	YES	YES	YES
City	285	285	285	285

*Standard errors in parentheses*.

*** *p < 0.01*,

** *p < 0.05*,

* *p < 0.1*.

In addition, new urbanization strengthens the economic cooperation and exchange between regions and makes technology spillover. The application and promotion of advanced technology among regions will reduce the cost of industrial pollution and the difficulty of pollution control. The improvement of technical level will reduce local environmental pollution and the overflow of local pollutants to surrounding cities. Therefore, technology spillover can reduce ecological damage and improve ecological benefits. However, upgrading the local industrial structure may produce a siphon effect and stimulate the gathering of more senior talents and enterprises, which has no obvious inhibitory effect on the environmental pollution of adjacent areas.

### Threshold Regression

In order to explore whether there is a nonlinear relationship between new urbanization and environmental pollution, we further analyze it through threshold regression. Previous empirical results verify that technology effect and industrial structure upgrading are two important intermediary variables. Therefore, we take technology effect and industrial structure upgrading as threshold variables to explore whether there is a nonlinear relationship between new urbanization and environmental pollution.

First, we need to determine the threshold and the number of thresholds. This paper uses the bootstrap method to test its significance 300 times. As shown in Table 11, there are double thresholds for technological effect and industrial structure upgrading. The thresholds for technological effect are 1.613 and 4.175, and the thresholds for industrial structure upgrading are 6.749 and 7.144.

**Table 11:** Threshold test.

**Variables**	**Test**	***F***	***P***	**10%**	**5%**	**1%**
TECH	Single	195.46	0.000	40.787	48.822	57.846
	Double	57.69	0.023	39.073	44.561	60.475
	Triple	38.00	0.753	124.978	132.197	145.445
INS	Single	68.52	0.000	32.917	38.243	48.464
	Double	38.07	0.040	30.785	37.219	48.966
	Triple	20.85	0.743	52.035	62.151	78.996

As shown in Table 12, when the level of new urbanization does not reach the first threshold of technological effect, the level of environmental pollution will decrease by 0.187 percentage points for every 1% increase in new urbanization. When the second threshold is exceeded, the level of environmental pollution will decrease by 0.454% points for every 1% increase in the level of new urbanization. It means that the inhibitory effect of new urbanization on environmental pollution will increase with the improvement of technological level. New urbanization promotes technological development, which can effectively reduce the difficulty of environmental governance. The optimization of the industrial technology development process promotes the application of green technology innovation, while ecological technology innovation with ecological preference significantly improves resource utilization efficiency and production efficiency, thus further improving the inhibition of environmental pollution.

**Table 12:** Threshold regression.

**Variables**	**(1)**	**(2)**
NUR (1.613> TECH)	−0.187^***^	
	(0.036)	
NUR (1.613≤TECH< 4.175)	−0.297^***^	
	(0.036)	
NUR (4.175≤TECH)	−0.454^***^	
	(0.035)	
NUR (6.749 > INS)		−0.252^***^
		(0.036)
NUR (6.749≤INS<7.144)		−0.316^***^
		(0.036)
NUR (7.144≤INS)		−0.423^***^
		(0.036)
HR	−0.011^***^	−0.010^***^
	(0.001)	(0.001)
LnGDP	−0.008	0.016^**^
	(0.008)	(0.008)
LnGDP2	0.001	−0.001
	(0.000)	(0.000)
OPEN	−0.000	0.000
	(0.000)	(0.000)
NBR	0.000^***^	0.000^***^
	(0.000)	(0.000)
Constant	0.078^**^	−0.036
	(0.039)	(0.039)
F	35.14^***^	33.52^***^

*Standard errors in parentheses*.

*** *p < 0.01*,

** *p < 0.05*.

When the threshold variable is industrial structure upgrading, the regression coefficient of the new urbanization to environmental pollution changes from −0.252 to −0.423 with the industrial structure upgrading. It shows that the inhibition of environmental pollution is strengthened. The development of new urbanization has gathered elements such as advanced technology, knowledge, and innovation, giving birth to the agglomeration and development of high-value-added industries. Some high polluting and backward sunset enterprises will withdraw from the market. The “extensive” development model will be transformed and upgraded. Industrial structure upgrading will promote non-agricultural investment, advanced technology, and production capacity in cities and improve resource utilization efficiency and environmental pollution control ability.

## Discussion

Through the empirical analysis above, this paper finds that similar to the research conclusions obtained by some scholars (10, 46, 47), the coefficients of new urbanization in various regression models are significantly negative, indicating that new urbanization has significantly reduced China's environmental pollution.

As a multi-dimensional urbanization development model, the new urbanization can restrain and alleviate environmental pollution through the economy, population, and society. Local environmental pollution may affect the environmental quality of surrounding areas under the influence of the natural geographical environment (61). Therefore, when discussing the impact of new urbanization on environmental pollution, environmental pollution's time inertia and spatial spillover effect should be considered. Secondly, considering the temporal and spatial correlation factors of environmental pollution, this paper discusses the impact of new urbanization on long-term and short-term environmental pollution. The results show that the new urbanization can improve the local environmental pollution in both the long term and the short term. However, new urbanization is still advancing in China, and the related policies are constantly being reformed and improved. Therefore, compared with the short term, the restraining effect of new urbanization on environmental pollution and the spatial spillover effect can only be fully exerted in the long term, which is related to (47). Therefore, the policymaking of new urbanization needs to maintain continuity.

We found that promoting population urbanization is an effective way for new urbanization to curb environmental pollution through empirical analysis. Whether long-term or short-term, population urbanization can inhibit and improve environmental pollution in surrounding areas. In the long term, social urbanization also has a spillover effect. Therefore, it shows that new urbanization has made significant contributions to environmental pollution by practicing the people-oriented concept. Here, the population urbanization we are talking about is not a simple population aggregation to urbanization and uncontrolled population growth. It emphasizes the coordination between the development of the urban population and the urban environment and improving urban residents' quality.

We analyze the inhibitory effect of population urbanization on environmental pollution for the following reasons. First, the concentration of the urban population is conducive to the centralized management of pollutants and improves the efficiency of pollutant treatment; economies of scale reduce the cost of public services and the unit cost of public transport. Second, the improvement of urban residents' awareness of environmental protection has put forward higher requirements for the living environment, increased citizens' social participation, and strengthened the supervision of government governance. Third, the spatial spillover effect of population on environmental pollution is mainly reflected in two aspects. On the one hand, improving local environmental quality reduces the spread of pollutants to surrounding cities. On the other hand, the population mobility between regions promotes the exchange of knowledge and technology. The learning effect between cities can effectively promote technology spillover, reduce the difficulty and cost of pollutant treatment, and improve the production efficiency of enterprises.

However, some scholars believe that the relationship between urbanization and environmental pollution may be nonlinear. Martínez-Zarzoso and Maruotti (2011) found an inverted U-shaped relationship between urbanization and CO2 emissions (62). He et al. (2017) studied a sample of 29 provinces in China from 1995 to 2013 and confirmed the Kuznets hypothesis (63). In order to test this, we tried to introduce the square term of new urbanization into the model.

We found that the primary term of new urbanization was not significant. In contrast, the square term passed the significance test, indicating an inverted U-shaped relationship between new urbanization and environmental pollution. By calculating the inflection point, however, we found that the new urbanization of the inflection point is 0.043. The new urbanization index minimum value is 0.053, which shows that the new urbanization has crossed the inflection point. Hence, the relationship between new urbanization and environmental pollution is linear. Therefore, promoting new urbanization development will significantly affect environmental pollution, indicating that our model setting is appropriate and the conclusions drawn are robust.

Like most articles, this study has some limitations. First, through the mediating effect model, this study finds that technological effect and industrial structure upgrading are two essential mechanisms for new urbanization to suppress environmental pollution. However, other variables play a role in this relationship, and future studies can continue to explore the interaction between other factors and environmental pollution. Second, this paper analyzes the impact of new urbanization on the environmental pollution level. However, new urbanization may have different effects on different polluting industries. Future studies can conduct comparative analyses by collecting relevant pollution data from polluting industries.

## Conclusion and Suggestion

New urbanization is an essential support for building ecological civilization and realizing sustainable development. Based on the panel data of 285 prefecture-level cities in China from 2003 to 2018, we apply the static and dynamic spatial Durbin model and mediating effect model to analyze the impact mechanism of new urbanization on environmental pollution from direct and indirect perspectives.

First, environmental pollution has obvious time inertia, and the pollution degree of this region is related to the environmental quality in the early stage. Therefore, to prevent the continuous deterioration of pollution, cities should take early prevention and early treatment of environmental pollution, strengthen environmental supervision of economic activities, especially industrial production, and regularly check whether the discharge of industrial enterprises exceeds the standard. Environmental pollution has a spatial spillover effect. The environmental pollution of the local area shows the phenomenon of infection in the surrounding area. Therefore, a regional joint prevention mechanism should be established under overall planning.

Second, new urbanization has a significant impact on environmental pollution. Dynamic spatial econometric analysis shows that, compared with the short-term, new urbanization has a more obvious inhibition effect on environmental pollution and a spatial spillover effect in the long term. Therefore, formulating and implementing a new urbanization strategy policy should ensure continuity, establish a scientific investment system for pollution control, and unswervingly implement environmental protection policies.

Third, population urbanization is essential in controlling environmental pollution and has a spillover effect. In the long run, social urbanization also has a spillover effect. The government should promote population mobility and the citizenization of the floating population. Specifically, the government should establish a unified registered residence system in urban and rural areas to reduce the differences in public services caused by the registered residence system, such as gradually abolishing the privileges attached to the household registration and improving residents' awareness of environmental protection and their participation in public utilities. Population mobility may lead to the population quality gradient imbalance. Therefore, the government also needs to strengthen the construction of human capital and improve the social security system.

Fourth, the quantile model verifies the difference in the impact of new urbanization on environmental pollution under different pollution levels. Therefore, promoting new urbanization in various regions should adapt to local conditions. For areas with relatively low pollution levels, the role of population and green urbanization should be given full play. Plant site selection, chimney design, and urban and industrial planning should be reasonable. They should be placed as far as possible in places with space, good air circulation, and relatively thin population density. The government should comprehensively deepen the disclosure of environmental information, especially the monitoring results of urban environmental quality and key pollution sources that are widely concerned. For severely polluted areas, it is necessary to change the pattern of economic development, increase financial transfer payments and technological input to severely polluted areas, and strengthen the design and improvement of compensation mechanisms for ecological protection.

Finally, technological effect and industrial structure upgrading are two mediating mechanisms of new urbanization to alleviate environmental pollution. The construction of new urbanization should focus on the research and development and promotion of environmental protection technologies and promote the technological upgrading of enterprises through incentive measures (tax exemption, green certification, etc.,), which will promote the replacement of dirty energy with clean energy. Strengthen cooperation and technology exchange among enterprises and social and environmental groups so that advanced technology flows to less developed areas. In addition, the promotion of new urbanization should focus on optimizing the spatial distribution of industries and adjusting the industrial structure. The government should encourage the development of advanced manufacturing, high-tech, and service industries with low resource consumption and high added value and promote the development of environmental protection industries. For emerging industries, especially information technology, new energy, and new materials, the government should remove unnecessary barriers when new businesses enter the market and promote the adjustment of the industrial structure toward the direction of advanced development.

## Data Availability Statement

The raw data supporting the conclusions of this article will be made available by the authors, without undue reservation.

## Author Contributions

YZ designed the conceptual framework of the methodology, drafted, and revised the manuscript. QC had the initial idea for the research and project administration. Both authors have read and agreed to the published version of the manuscript.

## Funding

This research was supported by the National Social Science Foundation of China (No. 18AZD003); Foundation of Humanities and Social Sciences of the Ministry of Education of China (No. 17YJA790102); Soft Science Research Program Project of He'nan Province (No. 212400410136).

## Conflict of Interest

The authors declare that the research was conducted in the absence of any commercial or financial relationships that could be construed as a potential conflict of interest.

## Publisher's Note

All claims expressed in this article are solely those of the authors and do not necessarily represent those of their affiliated organizations, or those of the publisher, the editors and the reviewers. Any product that may be evaluated in this article, or claim that may be made by its manufacturer, is not guaranteed or endorsed by the publisher.
